# Identification of high-risk age groups for cisplatin-induced nausea and vomiting in young patients with sarcoma

**DOI:** 10.1186/s40780-025-00490-x

**Published:** 2025-09-29

**Authors:** Azusa Soejima, Koki Hashimoto, Kazuyoshi Kawakami, Wataru Suzuki, Takeshi Aoyama, Keisuke Ae, Masakazu Yamaguchi

**Affiliations:** 1 Department of Pharmacy, Cancer Institute Hospital, Japanese Foundation for Cancer Research, Ariake, 3-8-31 Ariake Koto-ku, Tokyo, 135–8550 Japan; 2 Department of Orthopedic Oncology, Cancer Institute Hospital, Japanese Foundation for Cancer Research, Ariake, 3-8-31 Ariake Koto-ku, Tokyo, 135-8550 Japan; 3 Department of Clinical Pharmacology, School of Pharmacy, Tokyo University of Pharmacy and Life Sciences, 1432-1 Horinouchi, Hachioji, Tokyo, 192-0392 Japan

**Keywords:** Chemotherapy-induced nausea and vomiting (CINV), Risk factors, Sarcoma, Cisplatin, Children, Adolescent and young adult (AYA)

## Abstract

**Background:**

Young age has been reported as a patient-related risk factor for chemotherapy-induced nausea and vomiting (CINV). However, there have been few reports of the status of CINV limited to younger patients. In this study, CINV status after cisplatin administration was investigated in young patients with sarcoma.

**Methods:**

This retrospective, observational study included patients younger than 65 years with sarcoma who received cisplatin-containing chemotherapy at the Department of Orthopaedic Surgery, Cancer Institute Hospital Ariake, Japan, between January 2011 and December 2020. The evaluated endpoints were the complete response (CR) rate, defined as no emesis and no use of rescue antiemetics, and the no-vomiting rate, defined as no emesis regardless of rescue antiemetic use, in three age groups: children (< 15 years), adolescent and young adult (AYA; ≥15 to < 40 years), and middle age (MA; ≥40 to ≤ 64 years).

**Results:**

Twenty patients in the children group, 32 in the AYA group, and 19 in the MA group were included in the analysis. CR rates overall and in the delayed phase were 20% (4/20) in the children group, 28.1% (9/32) in the AYA group, and 42.1% (8/19) in the MA group. CR rates in the acute phase were 70% (14/20) in the children group, 78.1% (25/32) in the AYA group, and 94.7% (18/19) in the MA group. No-vomiting rates overall and in the delayed phase were 50.0% (10/20) in the children group, 59.4% (19/32) in the AYA group, and 78.9% (15/19) in the MA group. No-vomiting rates in the acute phase were 95.0% (19/20) in the children group, 96.9% (31/32) in the AYA group, and 100% (19/19) in the MA group.

**Conclusion:**

Even in patients younger than 65 years, the risk of CINV seemed higher in younger age groups.

**Trial registration:**

Retrospectively registered.

## Background

Nausea and vomiting, side effects of anticancer drugs, are among the most difficult side effects to control completely. It is known that the risk of nausea and vomiting is related to the emetogenic risk of anticancer agents, treatment-related risk factors, and patient-related risk factors. Although young age has been reported as a patient-related risk factor, the definition of young age varies from report to report [1, 2]. Young age includes children, and adolescents and young adults (AYAs). Control of nausea and vomiting is important for children and AYAs because they must balance schoolwork and work with treatment. However, there have been few reports investigating nausea and vomiting in that age group.

Osteosarcoma is the most common malignant bone tumor, accounting for approximately 56% of bone sarcomas, usually affecting children and AYAs, with a median age of 16 years [3, 4].

Treatment of osteosarcoma with surgery alone was associated with a poor 2-year disease-free survival rate of less than 20%. However, the addition of adjuvant chemotherapy has been shown to significantly prolong the survival rate to 66% [5]. Cisplatin (CDDP), methotrexate, doxorubicin, and ifosfamide have been shown to be effective as adjuvant chemotherapy agents [6].

Therefore, it is important to complete cisplatin therapy, a key drug for adjuvant chemotherapy, in the treatment of osteosarcoma. On the other hand, some patients have difficulty with nausea and vomiting in clinical practice because of the emetogenic risk factors of young age and cisplatin.

The purpose of this study was to identify the higher-risk age group among young people and to examine appropriate antiemetic therapy. In this study, the incidence of nausea and vomiting was investigated in patients under 65 years of age who received CDDP-containing chemotherapy for sarcoma, which commonly occur in young people.

## Methods

### Patients

Patients with sarcoma aged < 65 years who received chemotherapy including CDDP between January 2011 and March 2020 at the Department of Orthopedic Oncology, Cancer Institute Hospital, were included. The regimens included doxorubicin(60 mg/㎡) plus cisplatin (AP) and CDDP monotherapy, along with antiemetic agents (neurokinin-1 receptor antagonists, 5-hydroxytryptamine-3 receptor antagonists, and dexamethasone) recommended for highly emetogenic risk regimens by the Japanese Association for the Proper Use of Antiemetic Agents Guidelines.

Exclusion criteria were the following: (i) patients with a history of prior chemotherapy; (ii) patients receiving steroids other than inhaled steroids and topical steroids; (iii) patients with obvious emetic conditions such as brain metastases or severe gastrointestinal transit disorders; (iv) symptomatic patients with ascites or pleural effusions requiring therapeutic puncture; (v) patients with gastrointestinal transit disturbances such as gastropyloric stenosis or intestinal obstruction; (vi) patients with convulsive diseases requiring anticonvulsant therapy; (vii) patients with nausea and vomiting of Common Terminology Criteria for Adverse Events (CTCAE) Version 5.0 Grade 1 or higher at least 24 h prior to the start of chemotherapy and patients using drugs with potential antiemetic or emetic effects at least 48 h prior to chemotherapy; and (viii) patients receiving abdominal or pelvic radiation therapy within 6 days prior to chemotherapy.

### Methods

The following clinical information of the study subjects was obtained retrospectively from our electronic medical record system. Age, sex, weight, body surface area (BSA), Eastern Cooperative Oncology Group (ECOG) performance status (PS), whether the patient was receiving olanzapine prophylaxis, whether the patient was receiving rescue treatment with antiemetic drugs, tissue type, regimen, CDDP dose, estimated glomerular filtration rate (eGFR, mL/min/1.73 m^2^), corrected calcium (mg/dL), serum sodium (mmol/L), and onset of emesis. The eGFR for age ≥ 19 years was calculated as 194 × serum creatinine (Scr, mg/dL) − 1.094 × age (years) − 0.287 (× 0.738 for females). The eGFR for age 18 years and younger was calculated using the serum creatinine reference value (ref Cr) as 110.2 × (ref Cr/Scr) + 2.93 [7]. The ref Cr for boys was calculated using height (hereafter, Ht, m) as -1.259Ht5 + 7.815Ht4-18.57Ht3 + 21.39Ht2-11.71Ht + 2.628. The ref Cr for girls was also calculated using Ht as -4.536Ht5 + 27.16Ht4-63.47Ht3 + 72.43Ht2-40.06Ht + 8.778. Corrected calcium was calculated as serum calcium (mg/dL) + (4-serum albumin (g/dL)).

The observation period was from the start of CDDP administration on day 1 (also defined as “hour 0”) to the morning of day 6 (“hour 120”). The complete response (CR) rate (no emesis, no use of rescue antiemetics) and the no-vomiting rate (no emesis, regardless of rescue antiemetic use) were evaluated in three groups: children (< 15 years old), AYA (≥ 15 to < 40 years old), and middle age (MA; ≥40 to ≤64 years old). The acute phase was defined as 0 to 24 h after the start of chemotherapy, and the delayed phase was defined as 24 h to 120 h after the start of chemotherapy.

The dose of CDDP was 100 mg/m2 for CDDP monotherapy and 100 mg/m2 or 120 mg/m2 for AP therapy.

### Statistical analysis

CR and no-vomiting rates were compared among the three groups using Fisher’s exact test. Baseline patient characteristics (Table 1) were analyzed with the Kruskal–Wallis test for non-normally distributed continuous variables, ANOVA for normally distributed continuous variables, and the chi-squared test for categorical variables. All analyses were conducted using EZR (version 1.61; Saitama Medical Center, Jichi Medical University, Saitama, Japan), a freely available R-based software [8]. A p-value < 0.05 was considered statistically significant.

**Table 1:** Patients’ characteristics

Characteristics	Children (*n* = 20)	AYA (*n* = 32)	MA (*n* = 19)	*P* value
Age, years*	13 (6–14)	25 (15–37)	49 (40–63)	< 0.001
Sex, female, n (%)^†^	7 (35.0%)	12 (37.5%)	6 (31.6%)	0.9122
Body weight, kg^‡^	44.0 (21.7–63.0)	59.7 (38.9–87.5)	70.3 (47.6–102.7)	< 0.001
BSA, m^2‡^	1.41 (0.86–1.69)	1.67 (1.27–2.04)	1.79 (1.42–2.09)	< 0.001
ECOG performance status, n (%) 0 / 1 / 2^†^	16 (80.0%) / 3 (15.0%) / 1 (5.0%)	28 (87.5%) / 4 (12.5%) / 0 (0.0%)	17 (89.5%) / 2 (10.5%) / 0 (0.0%)	0.588
Olanzapine prophylactic oral administration, n (%)^†^	4 (20.0%)	8 (25.0%)	1 (5.3%)	0.206
Type of sarcoma, n (%)^†^				0.016
Osteosarcoma, UPS of bone	20 (100.0%)	29 (90.6%)	16 (84.2%)	
Soft tissue sarcoma	0 (0.0%)	0 (0.0%)	3 (15.8%)	
Others	0 (0.0%)	3 (9.4%)	0 (0.0%)	
Chemotherapy regimens, n (%) AP / CDDP^†^	20 (100%) / 0 (0.0%)	32 (100.0%) / 0 (0.0%)	14 (73.7%) / 5 (26.3%)	< 0.001
CDDP dose administered, mg/m^2‡^	100 (100–120)	100 (100–120)	100 (100–100)	0.012
Clinical Laboratory Values				
eGFR, mL/min/1.73 m^2^*	133.4 (95.6–244.3)	107.0 (53.7–158.8)	87.6 (62.9–135.4)	< 0.001
Corrected calcium, mg/dL*	9.9 (9.2–10.5)	9.45 (8.9–11)	9.5 (9.2–11.2)	0.003
Serum sodium, mmol/L*	139 (138–142)	140 (134–144)	140 (136–143)	0.117

Values are expressed as median (min-max) values

AP: doxorubicin + cisplatin, BSA: body surface area, CDDP: cisplatin, ECOG-PS: Eastern Cooperative Oncology Group Performance Status, eGFR: estimated glomerular filtration rate, UPS: undifferentiated pleomorphic sarcoma

Data are presented as number or mean ± SD, number (%). Statistical analyses were performed using the *Kruskal–Wallis test, ^†^Chi-squared test, or ^‡^Analysis of variance (ANOVA)

## Results

### Patients’ characteristics

**Fig. 1:**
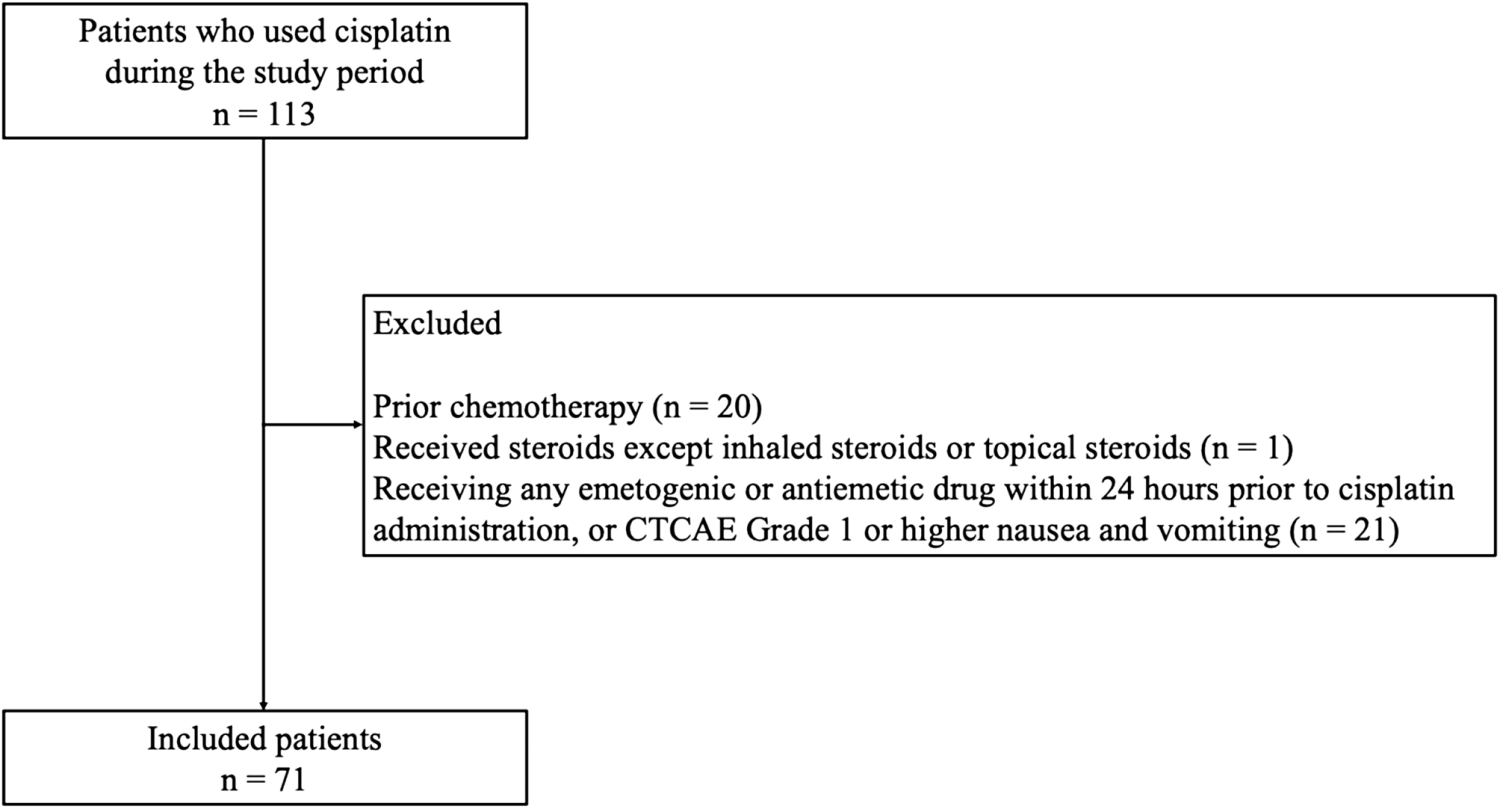
Patient flowchart and reasons for exclusion. The study included patients with sarcoma younger than 65 years of age treated with cisplatin between January 2011 and March 2020. Exclusion criteria were: (i) prior chemotherapy; (ii) receiving steroids other than inhaled or topical steroids; and (iii) receiving any emetogenic or antiemetic drug within 24 h prior to cisplatin administration, or CTCAE (Common Terminology Criteria for Adverse Events, Version 5.0) Grade 1 or higher nausea and vomiting

During the period of interest, 113 patients received chemotherapy including CDDP in our orthopedic surgery department, of which 71 patients were included in the analysis, excluding 42 patients who met the exclusion criteria (Fig. 1). All patients were administered neurokinin-1 receptor antagonists, 5-hydroxytryptamine-3 receptor antagonists, and dexamethasone. Olanzapine was administered prophylactically at a dose of 2.5 mg to 5 mg once daily after dinner or at bedtime for children and the AYA group, and at a dose of 5 mg once daily at bedtime for the MA group. The dosage was determined individually for each patient based on consultation between the physician and pharmacist. The dosage for children was set based on the report by Flank et al., with an initial dose of 0.10 mg/kg/dose as a reference [9]. The main patient characteristics considered when determining the dosage included the presence or absence of concomitant medications with sedative effects, the presence or absence of lower limb dysfunction due to the tumor, and the patient’s body size.

The patients’ characteristics are shown in Table 1. Twenty patients (28%) were in the children group, 32 (45%) in the AYA group, and 19 (27%) in the MA group. The proportion of females in each group was 35.0% (7/20) in the children group, 37.5% (12/32) in the AYA group, and 31.6% (6/19) in the MA group. Olanzapine prophylaxis was given to 20.0% (4/20) of the children patients, 25.0% (8/32) of the AYA patients, and 5.3% (1/19) of the MA patients. The proportion of patients who used rescue therapy between CDDP day 1 and day 6 was 80.0% (16/20) in the children group, 65.6% (21/32) in the AYA group, and 52.6% (10/19) in the MA group.

### CR rates by time period

**Fig. 2:**
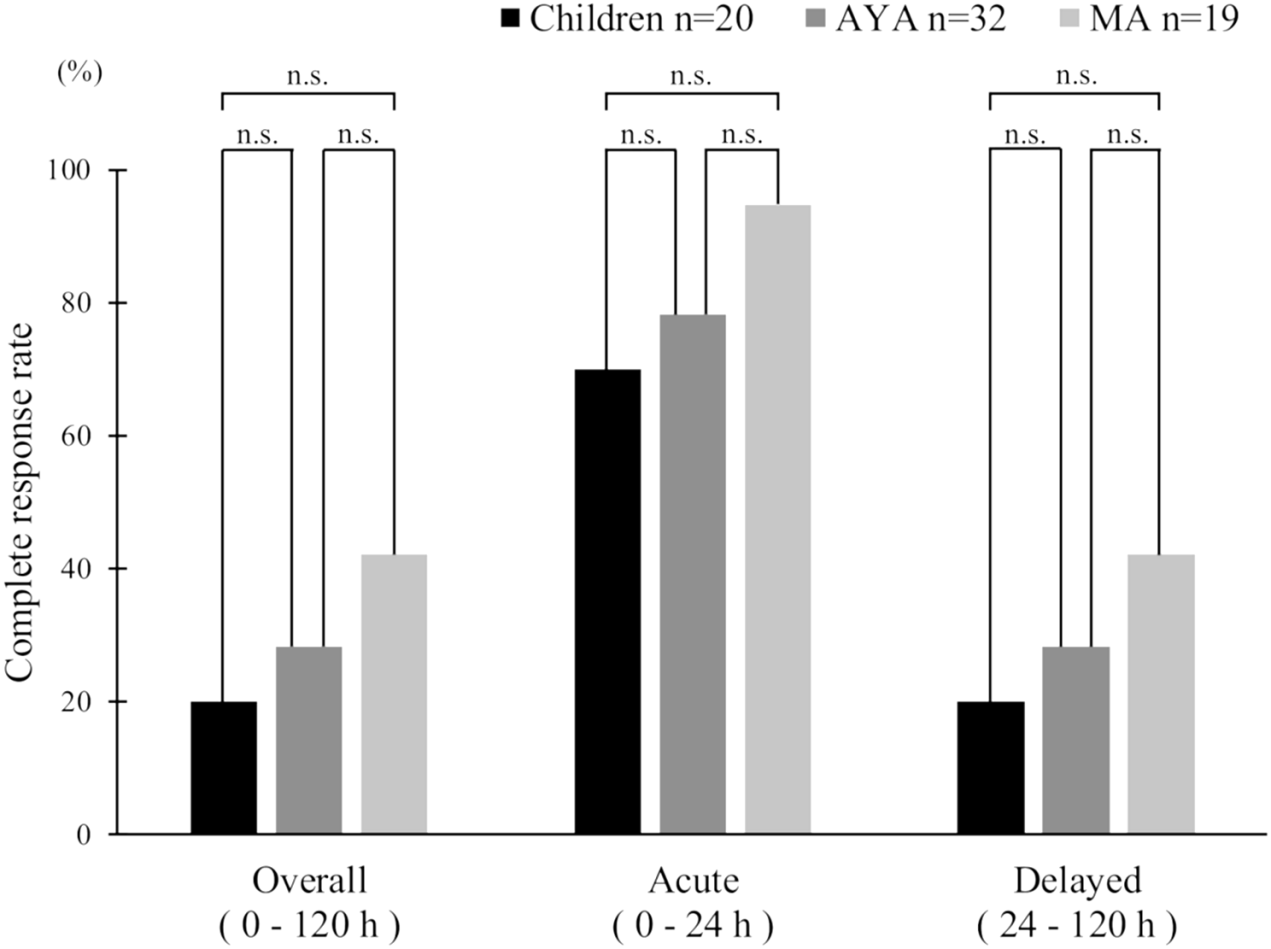
Complete response (CR) rate overall and by phase. This bar graph shows the percentage of patients achieving a CR 120 h after initiation of chemotherapy, with CR defined as no vomiting and no need for rescue medication. n.s., not significant

**Fig. 3:**
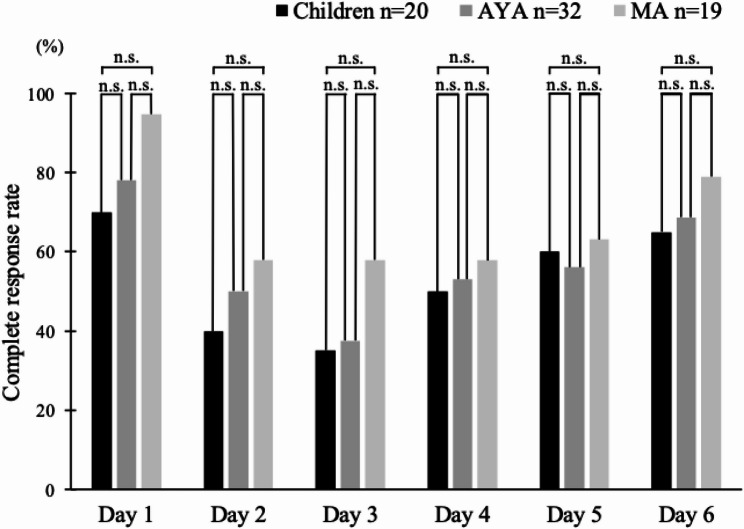
Complete response (CR) rate by day. This bar graph shows the percentage of patients who achieved a CR on individual days after cisplatin was initiated, with CR defined as no vomiting and no need for rescue medication. n.s., not significant

The CR rates during the evaluation period were as follows. The overall CR rate was 20% (4/20) in the children group, 28.1% (9/32) in the AYA group, and 42.1% (8/19) in the MA group; the CR rate in the acute phase was 70% (14/20) in the children group, 78.1% (25/32) in the AYA group, and 94.7% (18/19) in the MA group; and the CR rate in the delayed phase was 20% (4/20) in the children group, 28.1% (9/32) in the AYA group, and 42.1% (8/19) in the MA group (Fig. 2). There were no significant differences in CR rates between the overall, acute, and delayed phases in each group (Children, AYA, and MA). The CR rates for each time period from CDDP administration days 1 to 6 are shown in Fig. 3. The respective CR rates for CDDP days 1 through 6 were 70.0% (14/20), 40.0% (8/20), 35.0% (7/20), 50.0% (10/20), 60.0% (12/20), and 65.0% (13/20) in the children group, 78.1% (25/32), 78.1% (25/32), 50.0% (16/32), 37.5% (12/32), 53.1% (17/32), and 56.3% (18/32) in the AYA group, and 94.7% (18/19), 94.7% (18/19), 57.9% (11/19), 57.9% (11/19), 57.9% (11/19), and 63.2% (12/19) in the MA group There were no significant differences in CR rates for each day from CDDP administration days 1 to 6 among the three groups (Children, AYA, and MA).

### Non-vomiting rate by time period

**Fig. 4:**
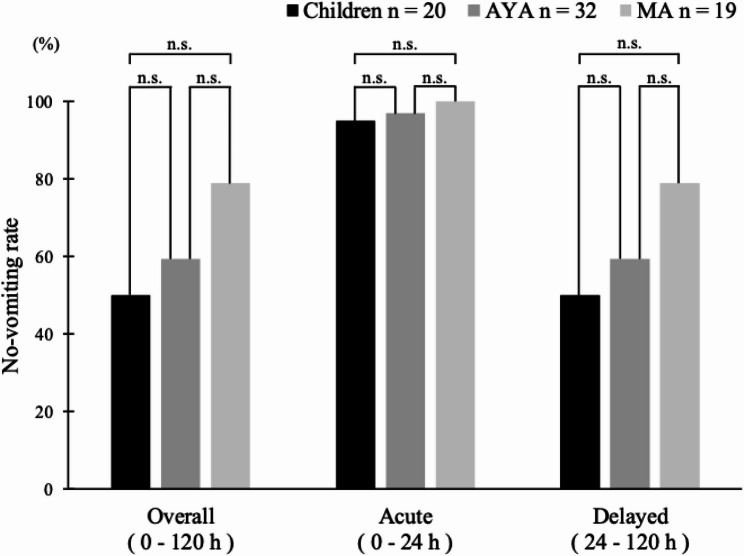
No-vomiting rate overall and by phase. This bar graph shows the percentage of patients achieving no-vomiting 120 h after initiation of chemotherapy, with no-vomiting defined as no emesis, regardless of rescue antiemetic use. There were no significant differences in the no-vomiting rates between the overall, acute, and delayed phases in each group. n.s., not significant

**Fig. 5:**
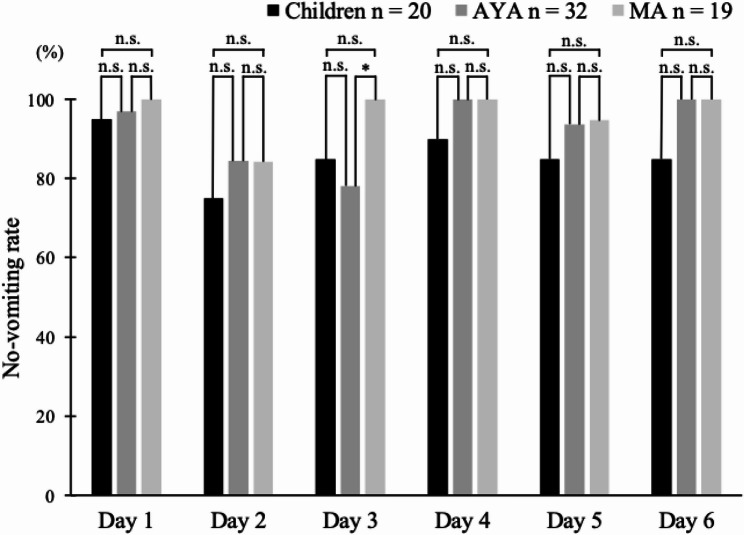
No-vomiting rate by day. This bar graph shows the percentage of patients who achieved no-vomiting on individual days after cisplatin was initiated, with no-vomiting defined as no emesis, regardless of rescue antiemetic use. The no-vomiting rate was significantly lower in the AYA group than in the MA group on day 3 (Fisher’s exact probability test *p* = 0.037). Statistical significance was defined as *p* < 0.05 (*); n.s., not significant

The no-vomiting rates during the evaluation period were as follows. The overall control rate was 50% (10/20) in the children group, 59.4% (19/32) in the AYA group, and 78.9% (15/19) in the MA group; the control rate in the acute phase was 95.0% (19/20) in the children group, 96.9% (31/32) in the AYA group, and 100% (19/19) in the MA group; and the control rate in the delayed phase was 50.0% (10/20) in the children group, 59.4% (19/32) in the AYA group, and 78.9% (15/19) in the MA group (Fig. 4). There were no significant differences in no-vomiting rates between the overall, acute, and delayed phases in each group (Children, AYA, and MA). The no-vomiting rates for each time period from CDDP administration days 1 to 6 are shown in Fig. 5. The respective control rates for CDDP days 1 through 6 were 95.0% (19/20), 75.0% (15/20), 85.0% (17/20), 90.0% (18/20), 85.0% (17/20), and 85.0% (17/20) in the children group; 96.9% (31/32), 84.4% (27/32), 78.1% (25/32), 100% (32/32), 93.8% (30/32), and 100% (32/32) in the AYA group; and 100% (19/19), 84.2% (16/19), 100% (19/19), 100% (19/19), 94.7% (18/19), and 100% (19/19) in the MA group. The no-vomiting rate was significantly lower in the AYA group than in the MA group on day 3 (Fisher’s exact probability test *p* = 0.037). There was no significant difference in no-vomiting rates at other time points.

The CR rate during the evaluation period for olanzapine-treated patients was 100% in all three groups in the acute phase, and 25% in the children group, 50% in the children and AYA groups and 100% in the MA group overall and in the delayed phase. The CR rates according to olanzapine use were as follows. In the children group, the CR rate was 25.0% (1/4) among olanzapine users and 12.5% (2/16) among non-users. In the AYA group, the CR rate was 50.0% (4/8) among users and 20.8% (5/24) among non-users. In the MA group, the CR rate was 100% (1/1) among the only user and 33.3% (6/18) among non-users.

### Percentage of rescue antiemetic use among patients who did not achieve CR

Among patients who did not achieve CR, the number of patients who had vomiting was 10 in the children group, 13 in the AYA group, and 4 in the MA group. Of these, the number of patients who also used rescue antiemetics was 10, 11, and 3, respectively. In this study, the rescue antiemetics administered included antihistamines (H₁ receptor antagonists), phenothiazine antipsychotics (dopamine D₂ receptor antagonists), benzodiazepine anxiolytics, and dopamine D₂ receptor antagonists/prokinetic agents. The choice of agent was made based on the clinical judgment of the attending physician, considering the patient’s symptoms and background.

### Percentage of treatment discontinuation among patients who did not achieve CR

In each group, among patients who did not achieve CR, none had a dose reduction of the second course of AP or CDDP therapy. However, treatment discontinuation was observed in 2 of 17 children (11.8%), 5 of 23 AYAs (21.7%), and 8 of 11 MAs (72.7%). The reasons for discontinuation were not related to CINV but included various clinical factors such as renal dysfunction and disease progression, which led to changes in the treatment plan.

## Discussion

This is the first report investigating the occurrence of CINV after cisplatin treatment in young patients with sarcoma, including children, stratified by age. The CR rate and no-vomiting rate were lower in the children group than in the AYA group and MA group at all phases, including the overall phase and the acute and delayed phases. Thus, in the group of patients younger than 65 years, CINV may be a problem in younger patients.

In all groups, the CR rate was lower in the delayed phase than in the acute phase. This result is similar to that reported by Suzuki et al. and suggests that control of CINV in the delayed phase is important even in young patients [10]. Furthermore, in this study, the CR rate tended to be lower in the acute phase in the children and AYA groups than in the MA group. This was similar to the study by Molassiotis et al., which included patients treated with highly and moderately emetogenic anticancer drugs, particularly in the acute phase [11]. The pattern of nausea onset was similar in all groups. In general, CINV tended to worsen on day 3 [12], and this trend was also observed in the children and AYA groups. In the children and AYA groups, it is necessary to consider measures to prevent nausea not only in the delayed phase but also during the acute phase. In a combined analysis of five studies, Mosa et al. reported that the risk of CINV decreased by 4% with each year increase in age [13]. In the present study, the CR rate and no-vomiting rate increased with increasing age in each age group, with similar results. Based on the above, younger age is considered to be a risk factor for CINV even in younger patients.

In addition to age, other risk factors for CINV have been reported in previous studies, including sex, alcohol consumption, and CDDP dosage [13–15]. In the patients in the present study, the proportion of females in each group was similar, and no electrolyte abnormalities that could contribute to nausea were observed in any of the groups. Because this study was conducted in children and AYAs, a history of alcohol consumption was not included in the survey. Minors were considered to have no history of alcohol consumption. In the children and AYA groups, some patients received CDDP at 120 mg/m², resulting in higher doses compared with other groups. However, eGFR was also higher in these younger patients, suggesting enhanced renal clearance of CDDP and a lower area under the curve (AUC), which could contribute to a higher CR rate. Therefore, the inclusion of patients receiving higher CDDP doses in the younger age groups likely had only a limited impact on the observed CR rates when renal function differences are considered. In addition, all patients in the children and AYA groups received AP therapy, whereas 26.3% of patients in the MA group received CDDP monotherapy. Although no direct comparative studies have been reported on the emetogenic risk of CDDP monotherapy versus CDDP combined with doxorubicin, doxorubicin is classified as a highly emetogenic agent. A randomized controlled trial by Edmonson et al. demonstrated that the combination of two highly emetogenic agents, such as doxorubicin and ifosfamide, resulted in a higher incidence of nausea compared with doxorubicin monotherapy [16]. Therefore, the combination of CDDP with highly emetogenic agents such as doxorubicin may potentially increase the risk of CINV. However, both regimens are classified as highly emetogenic, and at the time of the study, recommended prophylactic antiemetic therapy—including neurokinin-1 receptor antagonists, 5-hydroxytryptamine-3 receptor antagonists (all patients received palonosetron), and dexamethasone—was administered. Therefore, the impact on the current results is considered limited. Overall, while multiple factors may influence CINV, age appears to be the most significant determinant in this study.

All patients received palonosetron as prophylactic antiemetic therapy. There were no differences among the three groups in the proportion of patients who received aprepitant for 5 days, and in the doses and duration of 5-HT3 receptor antagonists and dexamethasone. Olanzapine prophylaxis was administered to less than 25% of patients in each group, but the proportion was relatively higher in the children and AYA groups. These results suggest that omitting prophylactic olanzapine administration, which has demonstrated efficacy against CINV, may further reduce the CR rate in children and AYA patients. In this study, the CR rate among olanzapine users was higher than that among non-users in each group: 25.0% vs. 12.5% in the children group, 50.0% vs. 20.8% in the AYA group, and 100% vs. 33.3% in the MA group. Although no statistical significance was observed due to the small sample size, these results suggest a trend toward improved CR rates with the addition of olanzapine. This finding is consistent with the results of the J-FORCE study, which demonstrated the efficacy of olanzapine in controlling CINV in patients receiving cisplatin-based chemotherapy [17]. In summary, differences in prophylactic antiemetic therapy appear to have had little impact on the current results.

The sample size of this study was limited to 71 cases. However, there have been few reports of CINV in young patients, including children, in previous studies. In a previous study of children and adolescents, 20 patients were included, and only 6 of them had osteosarcoma [18]. The results of the 71 young patients in the present study suggest that younger children and AYAs may be at risk for CINV, which is an important finding for future antiemetic therapy in young patients.

The results of this study indicated that, even among young cancer patients, the younger the age, the higher the risk of CINV. In particular, for children, control of nausea is important not only during the acute phase but also on days 2 and 3. In this study, the timing of CINV onset in children and AYA patients showed a pattern similar to that reported previously. In populations other than children, including adults, prophylactic antiemetic therapy with a four-drug combination including olanzapine is already recommended as standard for highly emetogenic regimens [17, 19]. In children as well, as indicated in the guidelines by Patel et al., a four-drug regimen including olanzapine is expected to achieve similar improvements as in adults when administered for regimens with a high emetogenic risk [20]. However, the optimal dose of olanzapine in children remains uncertain, and reported doses vary among studies [9, 21, 22]. Therefore, future prospective studies are needed to determine the appropriate pediatric dosing. Furthermore, attention must be paid to the safety profile. The safety profile of olanzapine includes reports of no clinically significant somnolence and reports of significant somnolence [23, 24]. One should consider “after dinner” dosing and dose reduction as a countermeasure in the event of somnolence [9, 17]. Because olanzapine takes 3–5 h to reach maximum blood levels, if administered after dinner, blood levels will peak while the patient is asleep. Thus, by implementing measures tailored to the patient’s level of somnolence, treatment can be conducted more safely.

Research on prophylactic antiemetic therapy is advancing. However, children are often not included in clinical trials. The results of the present study suggest that CINV may be a problem in younger patients, including children. We hope that the results of this study will lead to the development of appropriate prophylactic antiemetic therapy for younger patients.

## Conclusions

In patients younger than 65 years of age, the risk of CINV seems to be higher in younger age groups.

## Data Availability

No datasets were generated or analysed during the current study.

## Abbreviations

CINV: Chemotherapy-induced nausea and vomiting
CR: Complete response
AYA: Adolescent and young adult
MA: Middle age
CDDP: Cisplatin
AP: Doxorubicin plus cisplatin
CTCAE: Common Terminology Criteria for Adverse Events
BSA: Body surface area
ECOG: Eastern Cooperative Oncology Group
PS: Performance status
eGFR: Estimated glomerular filtration rate
Scr: Serum creatinine
ref Cr: Serum creatinine reference value
